# Multiple Audiometric Analysis in the Screening of Vestibular Schwannoma

**DOI:** 10.7759/cureus.21492

**Published:** 2022-01-22

**Authors:** Erika Celis-Aguilar, Alejandra Obeso-Pereda, Karla M Castro-Bórquez, Edgar Dehesa-Lopez, Alfredo Vega-Alarcon, Heloisa Coutinho-De Toledo

**Affiliations:** 1 Department of Otolaryngology, Head and Neck Surgery, Universidad Autonoma de Sinaloa, Culiacan, MEX; 2 Department of Internal Medicine, Universidad Autonoma de Sinaloa, Culiacan, MEX; 3 Department of Otolaryngology, Head and Neck Surgery, Instituto Nacional de Neurologia y Neurocirugia Manuel Velasco Suarez, Mexico City, MEX; 4 Department of Audiology, Hospital Medica Sur, Mexico City, MEX

**Keywords:** retrocochlear diseases, acoustic neuroma, sensorineural hearing loss, diagnostic tests, audiometry, vestibular schwannoma, asymmetric hearing loss

## Abstract

Introduction

Asymmetric sensorineural hearing loss is the main early symptom of retrocochlear lesions, hence its importance in screening for vestibular schwannomas. Currently, there is no consensus regarding its definition. The objective was to identify the audiometric pattern that would serve as a predictor for vestibular schwannoma in patients with asymmetric hearing loss.

Materials and methods

A cross-sectional study was conducted that included patients with asymmetric hearing loss attending a secondary care center and a tertiary care center. Clinical, audiometric and imaging (MRI with gadolinium) variables were collected. Asymmetric hearing loss was defined as a difference of 15 dB in one or more frequencies between both ears. The sensitivity, specificity, positive predictive value (PPV), negative predictive value (NPV) and accuracy of different audiometric patterns were analyzed.

Results

A total of 107 patients were studied and divided into two groups: group 1 without vestibular schwannoma (n=98); and group 2 with vestibular schwannoma (n=9). No significant difference in demographic characteristics or audiometric patterns was found in patients with and without vestibular schwannoma. The audiometric pattern with the best sensitivity as a screening test was a difference >20 dB in the 4,000 Hz frequency, with a sensitivity of 77.78%, specificity of 30.61%, PPV of 8.33%, NPV of 93.75% and accuracy of 34.50%.

Conclusion

The audiometric pattern with the best results was a difference >20 dB in the 4,000 Hz frequency range; however, patients with asymmetric hearing loss could not be differentiated from patients with retrocochlear lesions based only on audiometry. Asymmetrical hearing loss must be studied with MRI.

## Introduction

Asymmetric hearing loss is a difference in hearing loss between both ears, in which the contralateral ear might also be affected. Although hearing loss is the third most frequent cause of disability in some countries [1], there is currently no consensus regarding the definition of asymmetric hearing loss. Unfortunately, this lack of a standard definition makes the asymmetric hearing loss a controversial topic and this is aggravated by multiple definitions that have been previously published [2-15].

Furthermore, simple audiometry could be a potential early indicator of vestibular schwannoma (VS), becoming a simple and cost-effective tool for the screening of these tumors.

It is estimated that 2%-8% of patients with asymmetric hearing loss have retrocochlear lesions [15], this symptom could represent an early symptom, hence its importance in screening for VSs.

Asymmetric sensorineural hearing loss can be caused by damage to the cochlear hair cells, retrocochlear lesions and even cortical hearing loss. Additionally, we should consider cochlear nerve disease such as that produced by pontocerebellar angle lesions which can include meningiomas, petrous apex cholesteatomas, multiple sclerosis and, more commonly, VSs; this latter pathology causes mechanical compression of the cochlear nerve [16]. The incidence of VSs in the general population is 10 to 15 cases per million inhabitants per year [17,18].

VSs or acoustic neurinomas are benign, slow-growing tumors arising from the eighth cranial nerve. They are the most common tumors of the pontocerebellar angle and represent 6%-10% of intracranial tumors [10].

Actually, the main symptom of VS is sensorineural hearing loss which is usually asymmetric, gradual and high-tone hearing loss with greater asymmetry in the frequency range of 2-8 kHz [18].

The mechanism by which a VS causes hearing loss may not be attributed only to its size but are related to biomarkers, such as perilymph proteins and other specific subtypes of heat shock protein 70 (HSP70) [19].

Approximately 50% to 60% of VSs lead to gradual or abrupt hearing loss at high frequencies, with only 4% to 26% of patients presenting sudden hearing loss [20]. 

Gadolinium-enhanced nuclear magnetic resonance imaging (MRI) is the current diagnostic gold standard for retrocochlear lesions [5,21]. However, because the number of MRI exams needed on this group of patients is very large, and the number of schwannomas found is very small, screening algorithms have been developed to preserve and maximize resources [22].

The ideal screening test should be accurate and inexpensive, but to date, such a test does not exist in the initial diagnosis of schwannomas [23]. However, it is necessary to balance the possibility of not diagnosing pontocerebellar angle tumors vs. cost savings. Currently, there are multiple audiometric patterns described in the literature, reported to have sensitivities ranging from 88% to 90% and specificities from 30% to 57% [10,15,24]. Some diagnostic algorithms have also been described, combining the patient’s history and clinical symptoms with audiometric changes.

In our study, we sought to determine the audiometric pattern to be used as a screening test for VS.

## Materials and methods

The study design was a cross-sectional study. The location was multicenter. Consecutive patients were included from March 2009 to December 2010 at a tertiary care center and April 2016 to August 2018 at a secondary care center in a single otolaryngology clinic.

Subjects

The inclusion criteria were patients who attended the otorhinolaryngology service of a secondary care center and a tertiary care center with a diagnosis of asymmetric hearing loss by audiometry, defined as a difference of 15 dB in one or more frequencies between ears. After a diagnosis of asymmetric hearing loss, MRI was indicated. Only patients with MRI examinations were included.

The exclusion criteria were patients who did not consent to participate, patients with hearing loss of conductive origin, patients with retrocochlear lesions other than VSs, and patients with previous pontocerebellar angle surgery.

The elimination criteria were patients who had an incomplete audiometric study and/or absence of MRI.

Data on age, sex, tinnitus, vertigo, exposure to ototoxic drugs, trauma, ear surgery, noise exposure, stapedial reflexes, evoked potentials, MRI, otoacoustic emissions, and asymmetric hearing loss were collected.

Once the diagnosis of MRI was obtained, patients were ultimately compared and categorized into two groups: group 1 without VS (WOVS) and group 2 with VS (WVS). Clinical and demographic characteristics were described for Group 1 (WOVS) and Group 2 (WVS).

Definition of hearing loss

This study analyzed all possible definitions of asymmetric hearing loss and the most inclusive definition was chosen: a difference of 15 dB in one or more frequencies between ears.

All definitions available in the literature of asymmetric hearing loss were analyzed [2-15]. Calculations of each of these definitions were carried out for each patient. Also, a definition was added, which consisted of a ≥15 dB asymmetry at 4,000 Hz ( Modified 4000 Rule).

Statistical analysis

The data were analyzed with the SPSS statistical package, version 21.0 (SPSS, Chicago, IL, USA). Descriptive statistics were used with measures of central tendency and dispersion according to the distribution of the data. The sensitivity, specificity, positive predictive value (PPV), negative predictive value (NPV), positive likelihood ratio, negative likelihood ratio and accuracy were calculated. The data were compared using the χ^2^ test for categorical variables and Student’s t-test for continuous variables.

The definitions of asymmetric hearing loss described in the literature [2-15], were categorized as present or absent on each patient, and contingency tables were created for the calculation of diagnostic tests (sensitivity, specificity, PPV, NPV, accuracy). A receiver operator characteristic (ROC) curve was obtained to choose the best diagnostic test. Ethical approval was obtained from the Research Committee at our institution (Instituto Nacional de Neurología y Neurocirugía Manuel Velasco Suarez, no number assigned), informed consent was signed by all patients.

## Results

A total of 107 patients were included; 1 patient was excluded due to meningioma. Thus, 107 patients were ultimately included: group 1, without VS (WOVS) n=98 (91.6%) and group 2, with nine patients with VS (WVS) (8.4%).

Basal demographic characteristics were collected (Table 1) and analyzed without finding significant differences between the two groups. The mean age was 47.94±14.14 years old, and 68.2% (n=73) were women. The more frequently affected ear was the left ear in 54.2% (n=58), and the most frequently observed type of hearing loss was gradual hearing loss in 69.2% (n=74) of the cases. The mean age of group 1 (WOVS) was 47.86±14.1 years old, and the mean age of group 2 (WVS) was 48.89 ± 14.4 years old (p=0.145).

**Table 1:** Demographic characteristics of the population WOVS: Without Vestibular Schwannoma, WVS: With Vestibular Schwannoma.

Demographic characteristics			Groups						p
			WOVS.			WVS.			
			n	%		n		%	
Sex		Female	68	69.4			5	55.6	0.394
		Male	30	30.6			4	44.4	
Affected ear		Right	47	48.0			2	22.2	0.138
		Left	51	52.0			7	77.8	
Type of hearing loss		Progressive	66	68.8			8	88.9	0.205
		Sudden	30	31.3			1	11.1	
Time of progress in months (mean, standard deviation)			45.35 ± 91.66		47.11 ± 46.55				0.644
Headache	Negative		78	79.6			8	88.9	0.502
	Positive		20	20.4			1	11.1	
Tinnitus	Negative		5	5.2			0	0	0.485
	Positive		92	94.8			9	100	
Tinnitus side	Right		36	39.1			2	22.2	0.322
	Left		43	46.7			7	77.8	
	Bilateral		3	3.3			0	0	
Vertigo	Negative		34	34.7			5	55.6	0.213
	Positive		64	65.3			4	44.4	
Unsteadiness	Negative		34	34.7			2	22.2	0.449
	Positive		64	65.3			7	77.8	
Noise exposure		Negative	78	86.7			8	88.9	0.945
		Positive	11	12.2			1	8.3	
Ototoxic Drugs		Negative	78	85.7			9	100	0.478
		Positive	11	12.1			0	0	
Family history		Negative	80	83.3			7	77.8	0.672
		Positive	16	16.7			2	22.2	
Trauma		Negative	88	89.8			8	88.9	0.932
		Positive	10	10.2			1	11.1	
Otoscopy		Normal	92	93.9			9	100	0.445
		Abnormal	6	6.1			0	0	
Facial movement		Normal	88	89.8			8	88.9	0.932
		Abnormal	10	10.2			1	11.1	
Electronystagmography		Normal	21	32.3			1	11.1	0.192
(n=74)		Abnormal	44	67.7			8	89.9	
Otoacoustic emissions (n=12)		Normal	1	9.1			0	0	0.735
		Abnormal	10	90.9			1	100	
Brains stem auditory evoked potentials (ABR) (n=20)		Normal	19	100			0	0	<0.0001
		Abnormal	0	0			1	100	
Stapedius reflex (n=105)		Normal	59	60.8			3	37.5	0.197
		Abnormal	38	39.2			5	62.5	

In our study, we observed that the left ear was more frequently affected in both groups (group 1=52.0% vs. group 2=77.8%; p=0.1). Progressive hearing loss was also a prevalent clinical feature in both groups (88.9% vs. 68.8%; p=0.2). The duration of hearing loss progression was 45.3 in group 1 vs. 47.11 months in group 2 (p=0.644). The absence of vertigo was 55.6% vs. 34.7%; (p=0.21), respectively.

Tinnitus was present in most patients, documented in 94.8% (n=92) of group 1 and 100% (n=9) of group 2, with no significant difference between the groups (p=0.45). However, 100% of the patients with bilateral tinnitus belonged to group 1.

The causes of facial mobility disorders observed in group 1 (WOVS) were as follows: Bell’s facial paralysis (n=6), recurrent facial paralysis (n=1), Melkersson-Rosenthal syndrome (n=1), demyelinating disease (n=1) and idiopathic (n=1). The only case of facial mobility disorder documented in group 2 (WVS) occurred in a patient with schwannoma and a history of facial paralysis one year prior to diagnosis.

An abnormal otoscopy was documented in 6 cases, in order of frequency: due to myringosclerosis (n=2), opaque membrane (n=2), tympanic graft (n=1) and an external auditory canal osteoma (n=1).

As part of their diagnostic protocol, some patients underwent neuro-otological tests, the results of which were not significantly different between the two groups, except for auditory brainstem response (p ≤ 0.001), nevertheless the sample is small (Table 1).

The mean threshold in every frequency between both ears was analyzed, as well as the mean asymmetries of sensorineural hearing loss are represented in Table 2. None of the mean asymmetries of sensorineural hearing were significant (Table 2).

**Table 2:** Mean hearing thresholds and MASNHL Results are represented by mean ± standard deviation. WOVS: Without Vestibular Schwannoma, WVS: With Vestibular Schwannoma, MHT: Mean hearing thresholds, MASNHL: Mean Asymmetries of Sensorineural Hearing Loss *Difference between mean hearing thresholds of the affected ear and best ear

Frequencies	WOVS		WVS		WOVS	WVS	p
	MHT of affected ear (dB)	MHT of best ear (dB)	MHT of affected ear (dB)	MHT of best ear (dB)	MASNHL (dB)*	MASNHL (dB)*	
150 Hz	50.26 ± 29.58	17.22 ± 12.77	33.89 ± 21.03	17.22 ± 12.77	32.65 ± 30.60	16.66 ± 19.20	0.128
250 Hz	52.96 ±29.18	16.53 ± 10.70	40.00 ± 29.68	16.11 ± 9.93	36.42 ± 29.39	23.88 ± 27.01	0.221
500 Hz	56.07 ± 30.46	14.90 ± 9.73	40.56 ± 28.98	17.22 ± 10.34	41.17 ± 30.74	23.33 ± 23.84	0.094
1,000 Hz	56.48 ± 30.87	15.46 ± 10.84	57.22 ± 44.37	12.78 ± 9.71	41.12 ± 31.44	44.44 ± 43.40	0.770
2,000 Hz	54.74 ± 30.95	16.33 ± 12.85	65.56 ± 49.65	14.44 ± 12.10	38.41 ± 31.11	51.11 ± 52.36	0.275
4,000 Hz	60.66 ± 31.43	23.57 ± 17.14	76.67 ± 46.90	25.00 ± 17.32	37.19 ± 30.75	51.66 ± 44.15	0.197
8,000 Hz	71.30 ± 30.08	30.41 ± 22.49	84.44 ± 39.95	27.22 ± 25.13	39.43 ± 32.63	57.22 ± 49.44	0.139

The audiometric assessments performed in both groups were analyzed based on the audiometric patterns previously described in the literature, and no significant differences were found in any pattern (Table 3).

**Table 3:** Asymmetry analysis according to the different definitions in the literature *: Analysis accomplished by using the frequency 4,000 Hz instead of 3,000 Hz. Modified Rule 4000 (¥):≥15 dB asymmetry at 4,000 Hz. AAO-HNS: American Academy of Otolaryngology-Head and Neck Surgery AMCLASS - Audiogram Classification System - (&): ≥10 dB at any two neighboring frequencies Obholzer (A): ≥15 dB in two neighboring frequencies if better ear averaging 250 to 8,000 Hz is <30 dB. Obholzer (B): ≥20 dB in two neighboring frequencies if better ear averaging 250 to 8,000 Hz is >30 dB.

Asymmetry	Group 1 (WOVS)		Group 2 (WSV)		p
	n	%	n	%	
UK Department of Health	83	84.7	7	77.8	0.587
Sunderland	72	73.5	5	55.6	0.252
AAO-HNS*	74	75.5	6	66.7	0.599
Oxford	76	77.6	6	66.7	0.460
Seattle	75	76.5	6	66.7	0.509
Nashville	89	90.8	7	77.8	0.218
AMCLASS (&)	83	84.7	7	77.8	0.587
Modified Rule 4000¥	77	78.6	7	77.8	0.956
Rule 4000	68	69.4	7	77.8	0.599
Cueva	88	79.6	7	77.8	0.897
Mangham	82	83.7	7	77.8	0.651
Schlauch y Levine	67	68.4	5	55.6	0.433
Sheppard	75	76.5	6	66.7	0.509
Obholzer (A)	69	70.4	6	66.7	0.815
Obholzer (B)	31	31.6	5	55.6	0.146

The sensitivity and specificity of each audiometric pattern were measured, finding seven audiometric patterns with sensitivity of 77.78% (Table 4). The highest specificity was at a difference >20 dB in two adjacent frequencies if the average at 250 to 8,000 Hz was >30 dB in the better ear.

**Table 4:** Sensitivity and specificity analysis S: Sensitivity, SPE: Specificity, PPV: Positive predictive value, NPV: Negative predictive value, PLR: Positive likelihood ratio, NLR: Negative Likelihood ratio *: Analysis accomplished by using the frequency 4,000 Hz instead of 3,000 Hz Modified Rule 4000 (¥):≥15 dB asymmetry at 4,000 Hz AAO-HNS: American Academy of Otolaryngology-Head and Neck Surgery AMCLASS - Audiogram Classification System - (&): ≥10 dB at any two neighboring frequencies Obholzer (A): ≥15 dB in two neighboring frequencies if better ear averaging 250 to 8,000 Hz is <30 dB. Obholzer (B): ≥20 dB in two neighboring frequencies if better ear averaging 250 to 8,000 Hz is >30 dB.

Type of Asymmetry	S	SPE	PPV	NPV	PLR	NLR	Accuracy
UK Department of health	77.78%	15.31%	7.78%	88.24%	0.92	1.45	20.56%
Sunderland	55.56%	26.53%	6.49%	86.67%	0.76	1.68	28.97%
AAO-HNS*	66.67%	24.49%	7.50%	88.89%	0.88	1.36	28.04%
Oxford	66.67%	22.45%	7.32%	88.00%	0.86	1.48	26.17%
Seattle	66.67%	23.47%	7.41%	88.46%	0.87	1.42	27.10%
Nashville	77.78%	9.18%	7.29%	81.82%	0.86	2.42	14.95%
AMCLASS (&)	77.78%	15.31%	7.78%	88.24%	0.92	1.45	20.56%
Modified Rule of 4000(¥)	77.78%	21.43%	8.33%	91.30%	0.99	1.04	26.17%
Rule 4000	77.78%	30.61%	9.33%	93.75%	1.12	0.73	34.58%
Cueva	77.78%	20.41%	8.24%	90.91%	0.98	1.09	25.23%
Mangham	77.78%	16.33%	7.87%	88.89%	0.93	1.36	21.50%
Schlauch y Levine	55.56%	31.63%	6.94%	88.57%	0.81	1.41	33.64%
Sheppard	66.67%	23.47%	7.41%	88.46%	0.87	1.42	27.10%
Obholzer (A)	66.67%	29.59%	8.00%	90.62%	0.95	1.13	32.71%
Obholzer (B)	55.56%	68.37%	8.41%	94.37%	1.76	0.65	67.29%

**Figure 1:**
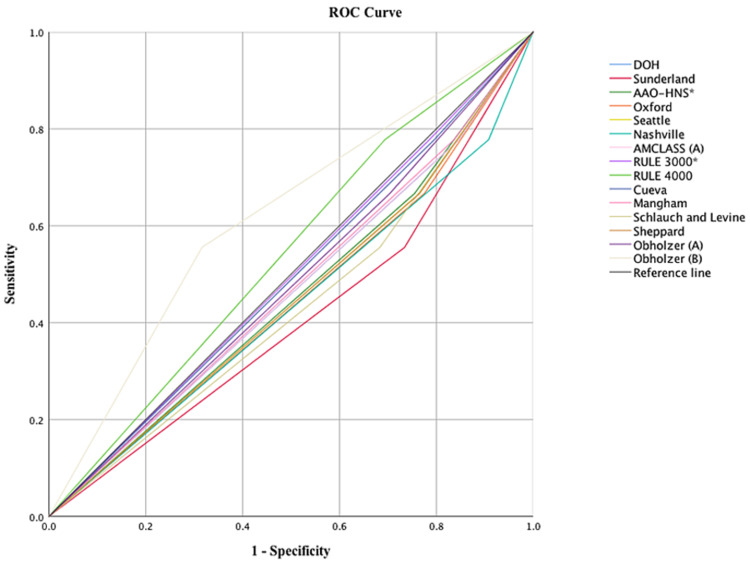
Receiver operating characteristic (ROC) according to the different definitions of asymmetric hearing loss

In the analysis of the receiver operating characteristic (ROC) curve (Figure 1), two audiometric patterns had a better performance: a difference ≥20 dB at 4,000 Hz, and a difference ≥20 dB at two adjacent frequencies when the average at 250 to 8,000 Hz was >30 dB in the better ear. The first pattern showed greater sensitivity and better specificity than the other audiometric patterns (sensitivity: 77.78%, specificity: 30.61%, PPV: 9.33%, NPV:93.75%); on the other hand, with greater specificity, there was a loss in sensitivity (sensitivity: 55.56%, specificity: 68.37%, PPV: 8.41%, NPV: 94.37%), respectively. Area Under the Curve (AUC) was 0.542 for the first audiometric pattern (4000 Hz rule) and 0.620 for the second audiometric pattern (Obholzer B). 

In summary, the pattern that showed the best result -as a screening test- was the 4,000 Hz rule, with a sensitivity of 77.78%, specificity of 30.61%, PPV of 9.33%, NPV of 93.75%, and accuracy of 34.58%.

## Discussion

In our study, no significant difference in demographic characteristics or audiometric patterns was found in patients with and without VS. This study represents a cross-sectional and multicenter study of asymmetrical hearing loss patients. There are multiple definitions of asymmetrical hearing loss to date (Table 5). Only nine patients with VS were included in this study; however, this corresponds to the usual prevalence of an asymmetric hearing loss population.

**Table 5:** Asymmetrical protocol parameters described in the literature AAO-HNS: American Academy of Otolaryngology-Head and Neck Surgery, AMCLASS: recommended asymmetry cutoff, dB: decibels, Hz: Hertz, S: Sensitivity, SPE: Specificity.

Asymmetrical parameters	S	Spe	Author
≥20 dB at any single frequency between 500 and 4,000 Hz	88%	31%	UK Department of health [2,3]
≥20 dB at any 2 neighboring frequencies	90%	39%	Sunderland [2,4]
≥15 dB between ears averaging 500 to 3,000 Hz	90%	57%	AAO-HNS [2]
≥15 dB between ears averaging 500 to 8,000 Hz	61%	62%	Oxford [4,6]
≥15 dB between ears averaging 1,000 to 8,000 Hz	89%	43%	Seattle [2]
≥15 dB at any single frequency between 500 and 4,000 Hz	83%	52%	Nashville [4,8]
≥10 dB at any 2 neighboring frequencies or ≥15 dB at any single frequency	82%	44%	AMCLASS [4,9]
≥15 dB asymmetry at 3,000 Hz	73%	76%	Rule 3000 [4,10]
≥20 dB asymmetry at 4,000 Hz	87%	62%	Rule 4000 [4,11]
≥15 dB at any 2 or more neighboring frequencies	83%	48%	Cueva [4,12]
≥10 dB between ears averaging 1,000 to 8,000 Hz	82%	44%	Mangham [4,13]
≥20 dB between ears averaging 1,000 to 8,000 Hz	91%	66%	Schlauch & Levine [4,11]
≥15 dB between ears averaging 250 to 8,000 Hz	85%	60%	Sheppard [4,14]
≥15 dB in 2 neighboring frequencies if better ear averaging 250 to 8,000 Hz is <30 dB or ≥20 dB in 2 neighboring frequencies if better ear averaging 250 to 8,000 Hz is >30 dB	91%	39%	Obholzer [2,15]

According to our results, the clinical characteristics (type of hearing loss, tinnitus, instability, family history, headache, otoscopy or facial mobility) did not allow us to identify patients with VS.

Tinnitus had a similar prevalence in both groups (p=0.322), interestingly, patients with VS presented with only unilateral tinnitus. Several studies have emphasized the importance of unilateral tinnitus for the diagnosis of retrocochlear lesions [23]. In contrast, Saliba et al. did not find tinnitus to be significantly related to VS [10].

Audiometry is considered a simple, economic, and easily available test without adverse effects, allowing us to consider it as an optimal screening test. This study had the aim to test all available definitions of asymmetric hearing loss in the literature, therefore, asymmetry ≥15 dB at any frequency was considered as an inclusion criterion; this definition could encompass all of the other definitions of asymmetric hearing loss. This definition has also been used as an inclusion criterion in previous studies [10,24]. High-frequency hearing loss could be the first diagnosis of a retrocochlear lesion, as corroborated by various studies [22-24]. Unfortunately, there are a number of other pathologies that may produce high-frequency hearing loss such as the aging ear and acoustic trauma.

The sensitivity, specificity, PPV and NPV of each of the definitions and frequencies were calculated, with the best performance found for asymmetry ≥20 dB at the 4,000 Hz frequency, which was better than the other definitions proposed in the literature.

Regarding the diagnostic algorithms of VSs, Saliba et al. [10] proposed a diagnostic approach consisting of performing MRI only in patients with asymmetric hearing loss of ≥15 dB at 3,000 Hz, known as the “3000 Hz rule,” with approximately 70% sensitivity and 70% specificity. In our center, unfortunately, this frequency is not measured routinely; however, the closest frequency is - 4,000 Hz - this frequency had higher parameters in ≥20 dB of difference between ears. This frequency (4,000 Hz) could be later named as the “4000 Hz rule” since it showed the best parameters as a diagnostic test (sensitivity: 77.78%, specificity: 30.61%, PPV: 9.33%, NPV: 93.75%).

A study conducted in Denmark by Gimsing [24] calculated the sensitivity and specificity of all of the definitions of asymmetry in the literature in a population of 199 patients with schwannoma and 225 patients without schwannoma who had undergone an MRI. They found a sensitivity of 73% to 100% with an average specificity of 50%.

Similarly, Obholzer et al. [15] performed an analysis of the sensitivity and specificity of each of the different audiometric criteria of asymmetric hearing loss for the diagnosis of retrocochlear lesions, using MRI as a gold standard. The authors concluded that asymmetric hearing loss of two adjacent frequencies >15 dB if the threshold of the better ear is ≤30 dB or asymmetry of 20 dB if the threshold of the better ear is >30 dB, with a sensitivity and specificity of 91% and 39%, respectively. This finding contrasted with the low sensitivity and specificity of 67% and 56%, respectively, found in our study. During our analysis, it was found that asymmetry of ≥20 dB at two adjacent frequencies when the average at 250 to 8,000 Hz was >30 dB in the better ear, showed the highest specificity of all (68.37%); however, the sensitivity was very low (55.56%), so it could not be considered an ideal screening test for VS. This high specificity has been previously published by other authors [25]. Unfortunately, in a screening test, the sensitivity needs to be prioritized.

Our results had low sensitivity and specificity in all the parameters studied. It is necessary to emphasize that the most relevant studies comparing the audiometry of patients with and without tumors were performed retrospectively based on MRI. These former studies were not prospective studies but case-control studies. This fact explains the paradoxical normal hearing in their control groups [10,23]. In contrast, our study relies on asymmetrical hearing loss as inclusion criteria and also represents one of the few cross-sectional studies available to date.

Unlike what has been described in another study that included asymmetry ≥15 dB in its logistic regression model as an independent predictor variable of the presence of VS [23], in our study, we analyzed the audiometry based on the definitions of asymmetric hearing described in the literature, and none of these definitions was independently related to increased risk of presenting with VS.

Strengths of this study rely on the multiple analysis of all the asymmetric hearing loss definitions available in the literature; additionally, we studied each frequency and its respective asymmetries. Since the audiometry did not show an adequate performance as a screening test, it relies on us, the clinicians, to indicate the MRI in any case of suspicion; nevertheless, the best definition in this study was the Rule of 4000.

It is important to consider these results since an undiagnosed schwannoma misses the possibility of being submitted on time to a procedure (radio-neurosurgery or neurosurgery) to preserve hearing and increase the quality of life of our patients.

The most recent study on MRI and asymmetric hearing loss, included 1059 patients, among those patients, there were only 16 patients with VS. The authors performed equally an analysis with the different definitions of asymmetric hearing loss. Overall, their results are similar to ours, but our sensitivity is lower since none of the definitions reached 100%. Additionally, the proportion of VS patients in Bhargava study is much lower than ours; which is explained due to the reduced incidence of the disease. Cross-sectional studies tend to have scant VS patients. 

The main limitation of this study is the low number of patients with VS; nonetheless, the occurrence of VS is 2%-8% [15] in patients with asymmetric hearing loss in a population-based study. Our study was performed on consecutive patients, so our numbers could portray the day-to-day cases of an otolaryngologic clinic. Since this is a study of only two hospitals, these data could not be extrapolated to a nationwide external validity sample; nevertheless, our numbers are similar to previously published data. More studies are needed to corroborate our results.

Till today, the MRI is the gold standard in the study of asymmetric hearing loss.

## Conclusions

Patients seen at a neurotology and otolaryngology center with asymmetric hearing loss could not be differentiated from patients with retrocochlear lesions based on audiometry alone. No significant difference in demographic characteristics or audiometric patterns was found in patients with and without VS. Clinical suspicion is recommended if there is asymmetry of at least 20 dB in the 4,000 Hz frequency. Asymmetrical hearing loss must be studied with MRI. More studies are needed to corroborate our results.

## References

[1] GBD 2016 Disease and Injury Incidence and Prevalence Collaborators (2017). Global, regional, and national incidence, prevalence, and years lived with disability for 328 diseases and injuries for 195 countries, 1990-2016: a systematic analysis for the Global Burden of Disease Study 2016. Lancet.

[2] Hentschel M, Scholte M, Steens S, Kunst H, Rovers M (2017). The diagnostic accuracy of non-imaging screening protocols for vestibular schwannoma in patients with asymmetrical hearing loss and/or unilateral audiovestibular dysfunction: a diagnostic review and meta-analysis. Clin Otolaryngol.

[3] Nouraei SA, Huys QJ, Chatrath P, Powles J, Harcourt JP (2007). Screening patients with sensorineural hearing loss for vestibular schwannoma using a Bayesian classifier. Clin Otolaryngol.

[4] Cheng TC, Wareing MJ (2012). Three-year ear, nose, and throat cross-sectional analysis of audiometric protocols for magnetic resonance imaging screening of acoustic tumors. Otolaryngol Head Neck Surg.

[5] Hojjat H, Svider PF, Davoodian P, Hong RS, Folbe AJ, Eloy JA, Shkoukani MA (2017). To image or not to image? A cost-effectiveness analysis of MRI for patients with asymmetric sensorineural hearing loss. Laryngoscope.

[6] (1995). Committee on Hearing and Equilibrium guidelines for the evaluation of hearing preservation in acoustic neuroma (vestibular schwannoma). Otolaryngol Head Neck Surg.

[7] Tolisano AM, Burgos RM, Lustik MB, Mitchell LA, Littlefield PD (2018). Asymmetric hearing loss prompting MRI referral in a military population: redefining audiometric criteria. Otolaryngol Head Neck Surg.

[8] Welling DB, Glasscock ME 3rd, Woods CI, Jackson CG (1990). Acoustic neuroma: a cost-effective approach. Otolaryngol Head Neck Surg.

[9] Margolis RH, Saly GL (2008). Asymmetric hearing loss: definition, validation, and prevalence. Otol Neurotol.

[10] Saliba I, Martineau G, Chagnon M (2009). Asymmetric hearing loss: rule 3,000 for screening vestibular schwannoma. Otol Neurotol.

[11] Schlauch RS, Levine S, Li Y, Haines S (1995). Evaluating hearing threshold differences between ears as a screen for acoustic neuroma. J Speech Hear Res.

[12] Cueva RA (2004). Auditory brainstem response versus magnetic resonance imaging for the evaluation of asymmetric sensorineural hearing loss. Laryngoscope.

[13] Mangham CA (1991). Hearing threshold difference between ears and risk of acoustic tumor. Otolaryngol Head Neck Surg.

[14] Sheppard IJ, Milford CA, Anslow P (1996). MRI in the detection of acoustic neuromas--a suggested protocol for screening. Clin Otolaryngol Allied Sci.

[15] Obholzer RJ, Rea PA, Harcourt JP (2004). Magnetic resonance imaging screening for vestibular schwannoma: analysis of published protocols. J Laryngol Otol.

[16] Kshettry VR, Hsieh JK, Ostrom QT, Kruchko C, Barnholtz-Sloan JS (2015). Incidence of vestibular schwannomas in the United States. J Neurooncol.

[17] Tos M, Stangerup SE, Cayé-Thomasen P, Tos T, Thomsen J (2004). What is the real incidence of vestibular schwannoma?. Arch Otolaryngol Head Neck Surg.

[18] Kotlarz JP, Eby TL, Borton TE (1992). Analysis of the efficiency of retrocochlear screening. Laryngoscope.

[19] Lassaletta L, Calvino M, Morales-Puebla JM, Lapunzina P, Rodriguez-de la Rosa L, Varela-Nieto I, Martinez-Glez V (2019). Biomarkers in vestibular schwannoma-associated hearing loss. Front Neurol.

[20] Matthies C, Samii M (1997). Management of 1000 vestibular schwannomas (acoustic neuromas): clinical presentation. Neurosurgery.

[21] Alexander TH, Harris JP (2013). Incidence of sudden sensorineural hearing loss. Otol Neurotol.

[22] Murphy MR, Selesnick SH (2002). Cost-effective diagnosis of acoustic neuromas: a philosophical, macroeconomic, and technological decision. Otolaryngol Head Neck Surg.

[23] Ruckenstein M, Cueva Cueva, Morrison P (1996). A prospective study of ABR and MRI in the screening of vestibular schwannomas. Am Journal Otol.

[24] Gimsing S (2010). Vestibular schwannoma: when to look for it?. J Laryngol Otol.

[25] Bhargava EK, Coyle P, Wong B, Masood A, Qayyum A (2019). To scan or not to scan-a cross-sectional analysis of the clinical efficacy and cost-effectiveness of audiometric protocols for magnetic resonance imaging screening of vestibular schwannomas. Otol Neurotol.

